# Inulin alleviates atherosclerosis through improving lipid metabolism, inflammation, and gut microbiota in ApoE-knockout mice: the short-chain is more efficacious

**DOI:** 10.3389/fphar.2024.1445528

**Published:** 2024-10-10

**Authors:** Kun Zhang, Yu Zeng, Jiawei Li, Yingchun Huang, Nan Zhang, Yue Gong, Kaihu Xiao, Jian Chen, Tiantian Chen, Haomin Qiu, Sisi Lei, Fei Yan, Chunhui Lang, Xudong Duan, Xianwen Dong

**Affiliations:** ^1^ Chongqing University Three Gorges Hospital, Chongqing Municipality Clinical Research Center for Geriatric Diseases, Chongqing, China; ^2^ School of Medicine, Chongqing University, Chongqing, China; ^3^ Chongqing Academy of Animal Sciences, Chongqing, China; ^4^ Department of Clinical Laboratory Medicine, Southwest Hospital, Third Military Medical University (Army Medical University), Chongqing, China; ^5^ Department of General Surgery, The First Affiliated Hospital of Henan University of Chinese Medicine, Zhengzhou, China; ^6^ College of Life Sciences, Anhui Agricultural University, Hefei, China; ^7^ Department of Biochemistry and Molecular Biology, Chongqing Medical University, Chongqing, China; ^8^ Chongqing Institute of Green and Intelligent Technology, Chinese Academy of Sciences, Chongqing, China

**Keywords:** atherosclerosis, gut microbiota, inulin, inflammation, intestinal barrier, lipid metabolism

## Abstract

**Introduction:**

Atherosclerosis (AS) is considered the underlying cause of many diseases, particularly cardiovascular and cerebrovascular diseases. Inulin, a type of fructan, has shown potential in improving atherosclerosis, although there are conflicting findings. It is hypothesized that the polymerization degree of inulin may largely influence its therapeutic effectiveness. Therefore, this study aimed to investigate the effects and mechanisms of short-chain and long-chain inulin in AS.

**Methods:**

ApoE^−/−^ mice fed a high fat diet (HFD) were used to establish an atherosclerosis model. These mice received daily oral administration of either short-chain or long-chain inulin for 12 weeks. Plasma lipid metabolism-related indices were measured using biochemical analysis, and plasma immunological indices were analyzed via ELISA. The aorta, aortic root regions, liver tissue, adipose tissue, and colon tissue were examined through various staining techniques, including ORO staining, hematoxylin and eosin staining, Alcian blue staining, and immunofluorescent or immunohistochemical assays. Microbiome analysis was conducted in the cecal content.

**Results:**

The results indicated that both short-chain and long-chain inulin substantially reduced the formation of atherosclerotic plaques. Inulin also improved plasma lipid concentrations and hepatic lipid metabolism, and partially alleviated both localized (atherosclerotic lesions) and systemic inflammation. Short-chain inulin was more effective than long-chain inulin in reducing atherosclerotic plaques formation, enhancing lipid metabolism and reducing inflammation. Additionally, both types of inulin showed similar effectiveness in enhancing intestinal epithelial barrier integrity, gut microbiota composition and functionality.

**Conclusion:**

These findings suggest that inulin has a protective role against atherosclerosis by enhancing lipid metabolism, reducing inflammation, and improving intestinal barrier and gut microbiota. As a dietary intervention, short-chain inulin is more effective than long-chain inulin, offering clinical implications for using inulin as a therapeutic agent for atherosclerosis.

## 1 Introduction

Atherosclerosis (AS) is the pivotal factor leading to coronary heart disease, stroke, and peripheral arterial diseases. (Ruiz-Leon et al., 2019). The formation of AS is an intricate biological process involving lipid deposition, endothelial cell damage, and inflammatory infiltration (Falk, 2006). Despite the availability of numerous medications targeting AS, the incidence and mortality rates remain alarmingly high (Libby, 2021). Thus, the novel therapeutic strategies and medication are urgency to develop.

Inulin, a natural polysaccharide, exhibits excellent health benefits in regulating lipid metabolism, gut microbiota, and the expression of inflammatory factors (Qin et al., 2023). For instance, in Italian healthy volunteers, adding inulin to pasta decreased the levels of total cholesterol/HDL-cholesterol ratio, triglycerides, and lipoprotein (Russo et al., 2008). Similarly, oral inulin supplementation significantly ameliorated total serum cholesterol and triglycerides in chronic kidney disease patients (Lai et al., 2020). These human studies suggest that inulin has beneficial effects on lipid metabolism, making it a potential strategy for atherosclerosis. Moreover, a study on ApoE^−/−^ mice found that inulin reduced atherosclerotic lesion areas (Rault-Nania et al., 2006). Conversely, studies in APOE*3-Leiden mice indicated that inulin did not reduce hypercholesterolemia or atherosclerosis development. Instead, it aggravated atherosclerosis by increasing plasma total cholesterol (Hoving et al., 2018a; Hoving et al., 2018b). Thus, the effect of inulin on atherosclerosis is inconsistency.

The inconsistent effects of inulin may be attributed to its characteristics, particularly its degree of polymerization (DP). Inulin is a kind of linear fructans and includes different degree of polymerization, including the native inulin (DP 2–60), oligofructose (DP < 10), and short-chain fructo-oligosaccharides (DP 2–4) (Hughes et al., 2022). Study in rats reported that the high polymerization degree (DP 24 and DP 15) inulin, but not low polymerization degree (DP 10) inulin, reduced the serum cholesterol and triglyceride levels, and liver triglyceride concentration (Han et al., 2013). However, fructans increased the IgA concentrations in the order DP4 > DP8 > DP16 but not DP23 in the rat cecal (Ito et al., 2011). Thus, the inulin degree of polymerization is critical for its’ function in lipid metabolism, immune and others. Moreover, the varying results of inulin might be related to its wide-ranging effects on lipid metabolism, inflammation, and gut microbiota. Inulin, as a prebiotic, can increase gut microbiota *Bifidobacterium* counts and other beneficial taxa (Han et al., 2013; Birkeland et al., 2020). As a polysaccharide, inulin can be fermented, producing short-chain fatty acids, which are critical for health (Birkeland et al., 2020). Additionally, as an immunomodulator, inulin can ameliorate both localized and systemic inflammation (Roy and Dhaneshwar, 2023). It’s clear that gut microbiota, short-chain fatty acids, and inflammation are known key factors mediating lipid metabolism and atherosclerosis development (Ohira et al., 2017). However, no study has systematically investigated the effects of different polymerization degrees of inulin on lipid metabolism, inflammation, and gut microbiota.

Thus, this study aims to explore the effects of different polymerization degrees of inulin on atherosclerosis and the underlying mechanisms in ApoE^−/−^ mice. Our results indicate that inulin plays a protective role against atherosclerosis by improving lipid metabolism, decreasing inflammation, enhancing the intestinal barrier, and modulating gut microbiota. Interestingly, short-chain inulin appears to be more efficacious than long-chain inulin.

## 2 Materials and methods

### 2.1 Inulin acquisition

Two types of inulin (BioDuly Co., Ltd., Shanghai, China) were used in this experiment based on their degree of polymerization. The average polymerization degrees of short-chain inulin (S) and long-chain inulin (L) are 9 and 25, respectively.

### 2.2 Animals and ethics statement

The animal study was approved by the Institutional Animal Care and Use Committee (IACUC) of Chongqing University (IACUC Issue No.: CQU-IACUC-RE-202310–001). Male *ApoE* ^*−/−*^ mice (7 weeks old, 20 ± 2 g) were purchased from GemPharmatech Co., Ltd. (Shanghai, China). The *ApoE* knock-out mouse (*ApoE* ^*−/−*^) is a well-established model for the study of the formation and progression of AS. The mice were randomly divided into four groups and acclimated for 1 week prior to gavage. All mice were fed with a normal chow diet (NCD) and water *ad libitum*, and housed under controlled conditions (22°C ± 2°C; 50%–60% humidity; 12-h light/dark periods). All procedures followed the institutional ethical guidelines for animal experiments.

### 2.3 Groups and treatment

After 1 week of acclimatization, mice were fed NCD or high fat diet (HFD) supplemented with or without diet inulin (5 g/kg/day) according to previous studies (Hughes et al., 2022; Wu et al., 2022). Thus, there are 4 groups: the negative control group (NCD + PBS), mice were fed with NCD and gavaged with 0.2 mL phosphate-buffered saline (PBS); the positive control group (HFD + PBS), mice were fed with HFD and gavaged with 0.2 mL PBS; the short-chain inulin group (HFD + S), mice were fed with HFD and gavaged with short-chain inulin (5 g/kg/day); the long-chain inulin group (HFD + L), mice were fed with HFD and gavaged with long-chain inulin (5 g/kg/day). Ten mice per group and the intervention period lasted 12 weeks.

### 2.4 Tissue acquisition

At the end of the experiment, mice were euthanized, and tissue samples were collected. Blood was collected in anti-coagulation EDTA tubes, and plasma was separated by centrifugation at 3,000 rpm at 4°C for 10 min and stored at −80°C. The aortas, hearts, and livers were collected and fixed with 4% paraformaldehyde fixative for Oil Red O (ORO) staining analysis. Colon tissues were fixed for Alcian blue staining analysis, white fat tissues were fixed for hematoxylin and eosin (H&E) staining analysis, and cecal contents were stored at −80°C for 16S rRNA sequence analysis.

### 2.5 Plasma biochemical and immunological analysis

Total triglyceride (TG), total cholesterol (TC), high-density lipoprotein cholesterol (HDL-C), and low-density lipoprotein cholesterol (LDL-C) contents in plasma were measured using standard commercial kits and the Chemray 240 automatic biochemical analyzer (Servicebio Technology Co., Ltd., Wuhan, China). Plasma inflammatory factors, interleukin 1β (IL-1β) and tumor necrosis factor-α (TNF-α), were determined by ELISA kits (Catalog Numbers 88–7,324 and 88–7,013, respectively, Thermo Scientific Inc., MA, United States). All experimental procedures were carried out according to the kit manual instructions.

### 2.6 Aortic lesion analysis

The heart and aorta were collected and fixed in 4% paraformaldehyde. The atherosclerotic burden was quantified using an en-face preparation of the whole aorta and cross-sections of the aortic root, both stained with Oil Red O (ORO). The red-stained areas indicated the lesion areas. The lesion area relative to the total tissue area was quantified using ImageJ software (ImageJ 1.53e, Wayne Rasband, National Institutes of Health, Bethesda, MD, United States). The regions of interest (ROI) in the aorta and aortic root were automatically outlined using ImageJ, with consistent anatomical landmarks used across all samples to ensure uniformity. The images were converted to grayscale, and a threshold was applied to differentiate the red-stained lesion areas from the non-stained areas. The threshold images were analyzed using the “Analyze Particles” function in ImageJ to calculate the area of the lesion regions, and the total tissue area within the ROI was also measured. The lesion area was expressed as a percentage of the total tissue area within the ROI to account for variations in section size and tissue orientation.

### 2.7 Morphologic and histology analysis

Liver sections were stained with ORO to indicate lipid deposition, reflecting liver lesions. The lesion area was analyzed using ImageJ software. Adipose tissue sections were stained with hematoxylin and eosin (H&E) to observe adipocyte size and morphology. Adipocyte diameter was measured using ImageJ. The frozen sections of colon tissue were stained with Alcian blue. The blue-stained area represented the mucus layer, reflecting the mice’s intestinal barrier function. The blue area in the colon tissue sections was quantified by ImageJ.

### 2.8 Immunofluorescent and immunohistochemical assay

For immunofluorescent assays, frozen sections of aortic root or colon tissues were washed with PBS and blocked with 5% BSA for 30 min. They were then incubated with primary antibodies against intercellular adhesion molecule-1 (ICAM-1; 1:200; Santa Cruz Biotechnology, Inc., Dallas, TX, United States), CD68 (CD68; 1:400; Abcam Inc., Cambridge, United Kingdom), zonula occludens-1 (ZO-1; 1:100; Abcam Inc., Cambridge, United Kingdom), or occludin (1:100; Abcam Inc., Cambridge, United Kingdom). After overnight incubation, sections were incubated with secondary antibodies for 1 h and counterstained with DAPI (Beyotime Co., Ltd., Shanghai, China). Fluorescence images were obtained using a laser scanning confocal microscope. For immunohistochemical assays, sections of the aortic root were treated as previously described. After the endogenous peroxidase activity had been inhibited by 3% hydrogen peroxide for 25 min, sections were incubated with vascular cell adhesion protein 1 (VCAM-1; 1:200; Santa Cruz Biotechnology Inc., Dallas, TX, United States) overnight at 4°C. The sections were incubated with secondary antibody (HRP-labeled) for 1 h, and then diaminobenzidine was used to express the target area and the nucleus was counterstained with hematoxylin. Images were observed using an optical microscope. The positive staining area was measured by ImageJ and showed as a percentage of the total area.

### 2.9 Cecal content DNA extraction and 16S rRNA sequencing analysis

Fresh cecal contents were collected and immediately frozen in liquid nitrogen. Then samples were further explored the 16S rRNA sequence and bioinformatic analysis by Beijing Allwegene Gene Technology Co., Ltd., (Beijing, China). The primarily analyzed methods described by previous studies (Chen et al., 2018; Zhang et al., 2022). Briefly, bacterial DNA was extracted from cecal content with Qiagen DNeasy PowerSoil kit. The variable V3-V4 (338F-806R) region of the 16S rRNA gene was amplified using the primers (Forward: 5′-TCGTCGG CAGCGTCAGATGT GTATAAGAGACAGCCTACGGGNGGCWGCAG-3′, Reverse: 5′-GTCTCGTGGG CTCGGAGATGTGTATAAGAGACAGGACTACHVGGGTATCTAATCC-3′). The qualitied PCR products were further sequenced using the Miseq platform. Then the biological information analysis was performed based on the qualified data. Sequences were clustered into operational units (OTUs) at a similarity level of 97% to calculate richness and diversity indices. The representative sequences of each OTUs were screened for classified annotation at the phylum and genus levels. Alpha-diversity analysis was performed using QIIME (v1.8.0), including Chao1, Simpson, and Shannon indices. The analysis of beta diversity included principal component analysis (PCA) and principal coordinate analysis (PCoA) of the gut microbiota was examined the dissimilarities of the microbial communities among the groups. The non-metric multidimensional scaling (NMDS) of the gut microbiota was further analyzed based on Braye-Curtis similarities. Linear discriminant analysis (LDA) effect size (LEfSe) was performed on the different microbial features to identify the predominant bacteria with LDA score greater than 4.0.

### 2.10 RNA isolation and quantitative real-time PCR analysis

Total RNA was isolated from liver tissue using Trizol reagent (Thermo Scientific Inc., MA, United States) and reverse transcribed with the PrimeScript RNA Transcription Reagent Kit with gDNA Eraser (TaKaRa Inc., Tokyo, Japan). Real-time quantitative PCR (RT-qPCR) was performed using SYBR Green with a total volume of 20 µL. The reaction mixture included 1 µL DNA, 1 µL each of forward and reverse primers (10 µM each), 7 µL water, and 10 µL TB Green Premix Ex Taq II (Tli RNaseH Plus). All samples were repeated in triplicate and the relative expression of target gene was calculated using the ΔΔ-Ct method with GAPDH as the house keeping gene. All primer sequences are listed in Table 1.

**TABLE 1:** Primer sequences used for quantitative real-time PCR analyses.

Genes	Forward (5′-3′)	Reverse (5′-3′)
ABCA1	TGG​GCT​CCT​CCC​TGT​TTT​TG	GTC​ACT​TTC​ATG​GTC​GCT​GC
ABCG1	CTA​CGG​CTT​GGA​CCG​AGA​AG	ACC​TCT​CAG​CCC​GGA​TTT​TG
ABCG5	CCT​GCT​GAG​GCG​AGT​AAC​AA	TGG​CAC​CCA​CAA​GCT​GAT​AG
CYP7A1	CTG​GGG​GAT​TGC​TGT​GGT​AG	GCA​CAG​CCC​AGG​TAT​GGA​AT
PCSK9	ATC​ACC​GAC​TTC​AAC​AGC​GT	GCC​CTT​CCC​TTG​ACA​GTT​GA
SREBP1	GCC​ATC​GAC​TAC​ATC​CGC​TT	GTC​TCC​ACC​ACT​TCG​GGT​TT
GAPDH	GTA​TGA​CTC​CAC​TCA​CGG​CA	GAT​GTT​AGT​GGG​GTC​TCG​CT

### 2.11 Statistical analysis

Statistical analyses were performed using GraphPad Prism 7.0 (GraphPad Software), and all data were expressed as mean ± SD. For normally distributed data, one-way analysis of variance (ANOVA) was used to analyze the statistical differences between the groups. Subsequently, the Tukey *post hoc* test was used for multiple comparisons. For data that did not conform to a normal distribution, Kruskal-Wallis test was assessed first, followed by Dunn’s multiple comparison test. All *P* values were two-tailed, and a value of *P* < 0.05 was considered significant.

## 3 Results

### 3.1 Inulin alleviated atherosclerotic progression

**FIGURE 1:**
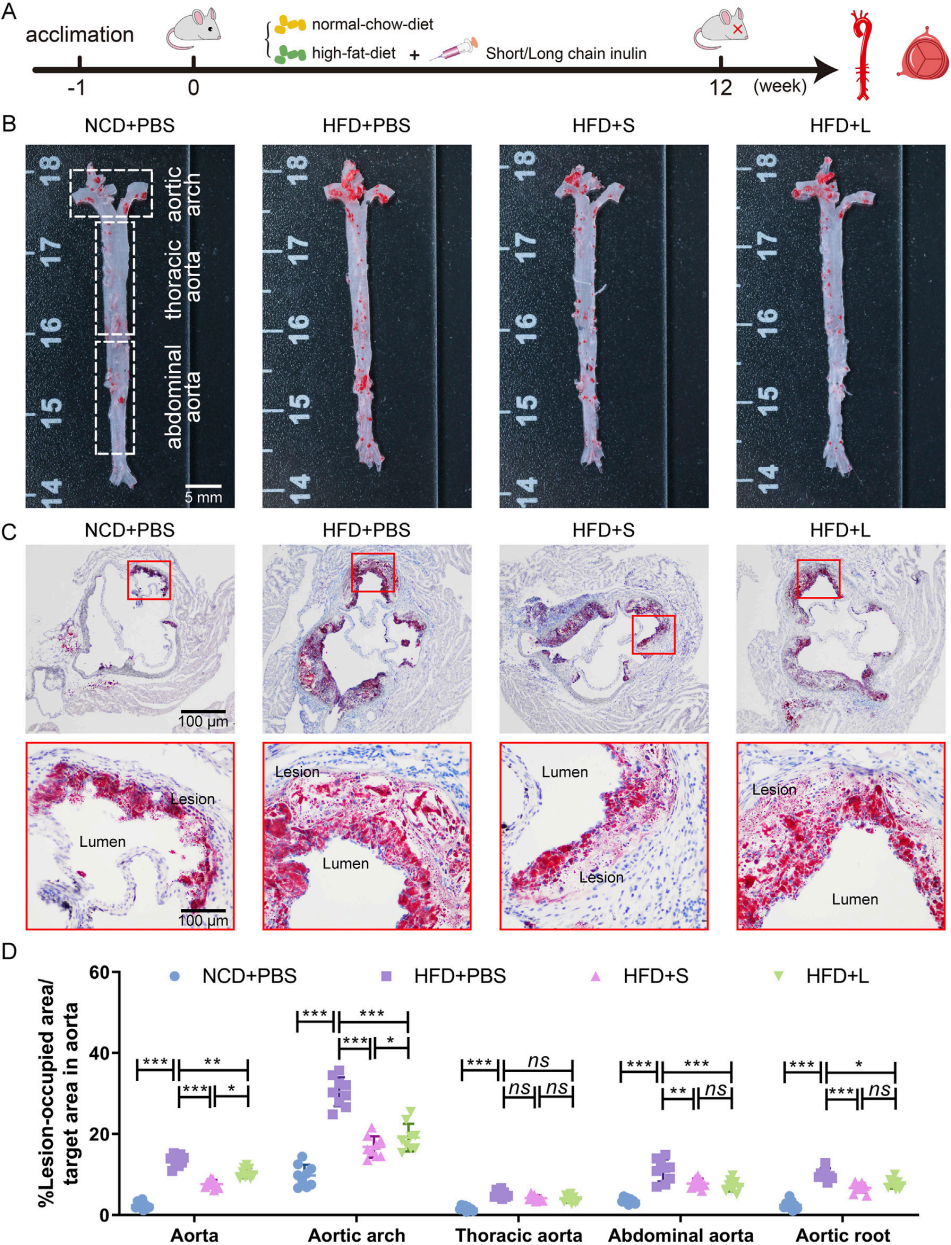
Inulin administration alleviated the progression of atherosclerosis in *ApoE* ^−/−^ mice. **(A)** Schematic illustration of the experimental design. **(B)** Representative images of plaque lesions in the whole area of the aorta stained with Oil Red O (ORO) staining. **(C)** Representative images of ORO staining of the aorta root sections. **(D)** Quantitative analysis of lesion area in aorta, aortic arch, thoracic aorta, abdominal aorta, and aorta root sections. Data were shown as mean ± SD. *n* = 9. Significances were determined by one-way analysis of variance (ANOVA), followed by *post hoc* pairwise comparisons with the Tukey honest significant difference. *ns* (no significance), *P* < 0.05 (*), *P* < 0.01 (**), or *P* < 0.001 (***).

*ApoE* ^−/−^ mice were fed a HFD supplemented with either short-chain or long-chain inulin daily for 12 weeks. After this period, the mice were sacrificed, and the extent of atherosclerosis was analyzed (Figure 1A). ORO staining revealed that HFD significantly increased aortic plaque size compared to the normal chow diet (NCD) cohort, whereas inulin supplementation reduced the progression of atherosclerotic lesions (Figures 1B, D). The aortic architecture could be further delineated into salient segments: the aortic arch, thoracic aorta and abdominal aorta. Inulin diminished atherosclerotic plaque within the aortic arch and abdominal aorta but not in the thoracic aorta (Figures 1B, D). A deeper examination probed into the aortic root lesions was further analyzed. Similarly, inulin incorporation profoundly attenuated the HFD-induced atherosclerotic expanse manifestations at the aortic root (Figures 1C, D). In comparison to long-chain inulin, the short-chain was more efficacy in attenuating the HFD-induced atherosclerotic lesion, especially in the aortic arch. These results demonstrated that HFD could induce atherosclerosis in the aorta of *ApoE* ^−/−^ mouse, while oral supplementation with inulin could significantly alleviated the progression of atherosclerotic plaques. Notably, short-chain inulin demonstrated more pronounced anti-atherosclerotic efficacy compared to the long-chain inulin.

### 3.2 Inulin modulated lipid metabolism homeostasis

**FIGURE 2:**
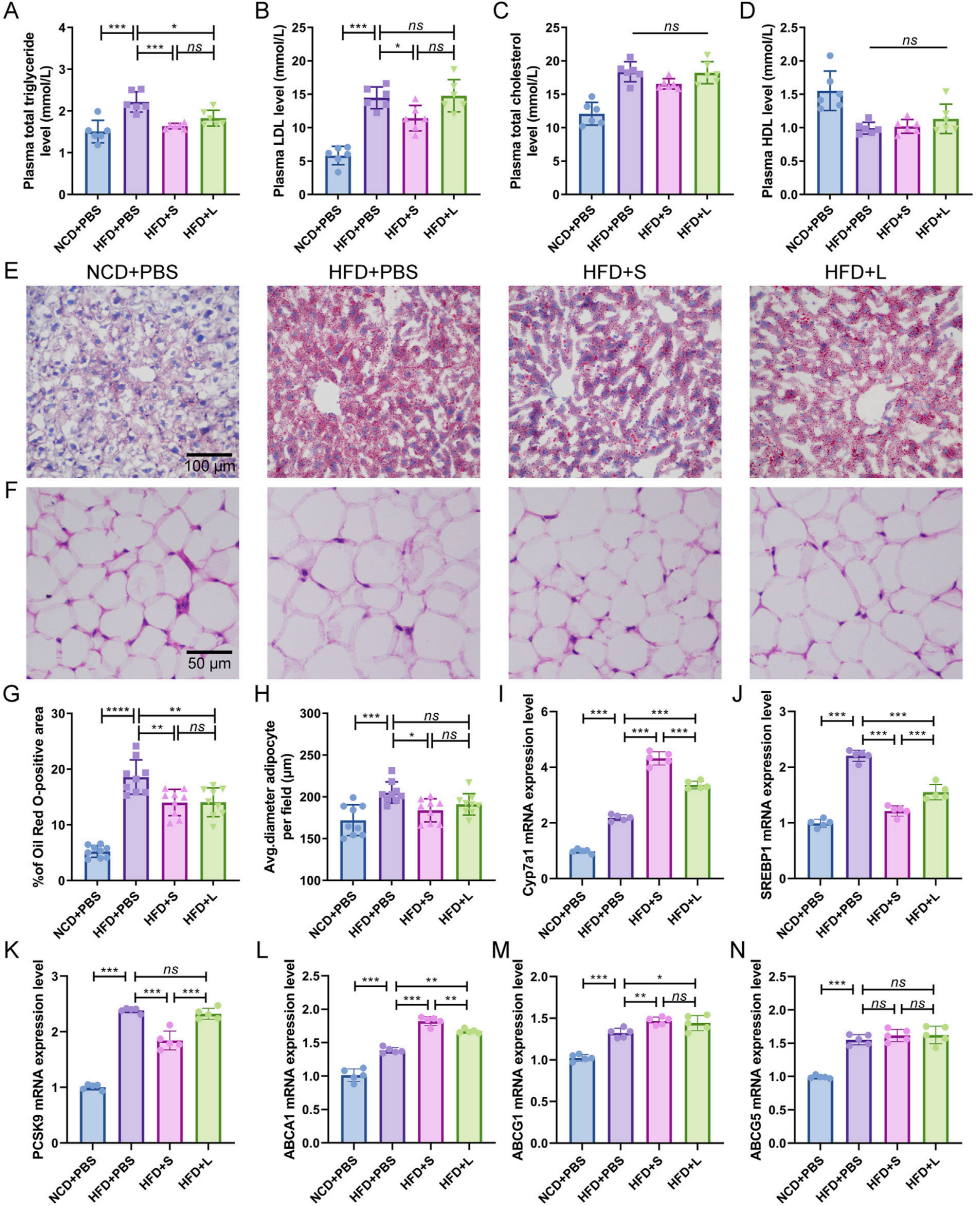
Inulin supplementation could improve lipid metabolism in *ApoE* ^−/−^ mice. **(A)** Plasma total triglyceride. **(B)** Plasma low-density lipoprotein (LDL). **(C)** Plasma total cholesterol. **(D)** Plasma High-density lipoprotein (HDL). **(E)** Representative images of Oil Red O (ORO) staining of liver sections. **(F)** Representative images of H&E staining of adipocytes. **(G)** Percentage of ORO positive area was calculated from **(E)** by ImageJ software. **(H)** Quantitative analysis of adipocyte size from **(F)** by ImageJ software. **(I–N)** Representative gene expression levels for Cyp7a1, SREBP1, PCSK9, ABCA1, ABCG1, and ABCG5 in the liver were quantified. Data were shown as mean ± SD. *n* = 5 to 9. Significances were determined by one-way analysis of variance (ANOVA), followed by *post hoc* pairwise comparisons with the Tukey honest significant difference. *ns* (no significance), *P* < 0.05 (*), *P* < 0.01 (**), *P* < 0.001 (***).

Atherosclerotic progression is tightly regulated by lipid metabolism. Consequently, the effect of inulin on plasma lipid concentration and hepatic lipid were analyzed. In comparison to the NCD group, the HFD induction increased or tended to increase the plasma triglyceride, LDL and total cholesterol concentrations while had a tendency to decrease HDL concentration (Figures 2A–D). Intriguingly, compared to the HFD treatment, both short-chain and long-chain inulin decreased the plasma triglyceride concentration (Figure 2A), only the short-chain inulin decreased plasma LDL concentration (Figure 2B). However, inulin supplementation did not markedly influence plasma total cholesterol concentrations (Figure 2C) or HDL concentrations (Figure 2D). Hepatic histological ORO staining analyses showed that inulin treatment diminished lipid accretion in liver tissue (Figures 2E, G). Moreover, the result also indicated that the white adipocytes in the inulin group were notably smaller compared to those in the HFD group (Figures 2F, H). To further understand the mechanism of inulin in lipid metabolism, the gene expression levels of Cyp7a1, SREBP1, PCSK9, ABCA1, ABCG1 and ABCG5 in the liver were quantified. The results showed that HFD induction increased all these genes expression level compared with the NCD group (Figures 2I–N). Interestingly, the inulin treatment further increased the mRNA expression of Cyp7a1, ABCA1 and ABCG1, while decreased the mRNA expression of SREBP1and PCSK9 (Figures 2I–M). The two-type inulin showed the same tendance but the short-chain inulin was more effective (Figures 2I–M).

### 3.3 Inulin ameliorate localized and systemic inflammation

**FIGURE 3:**
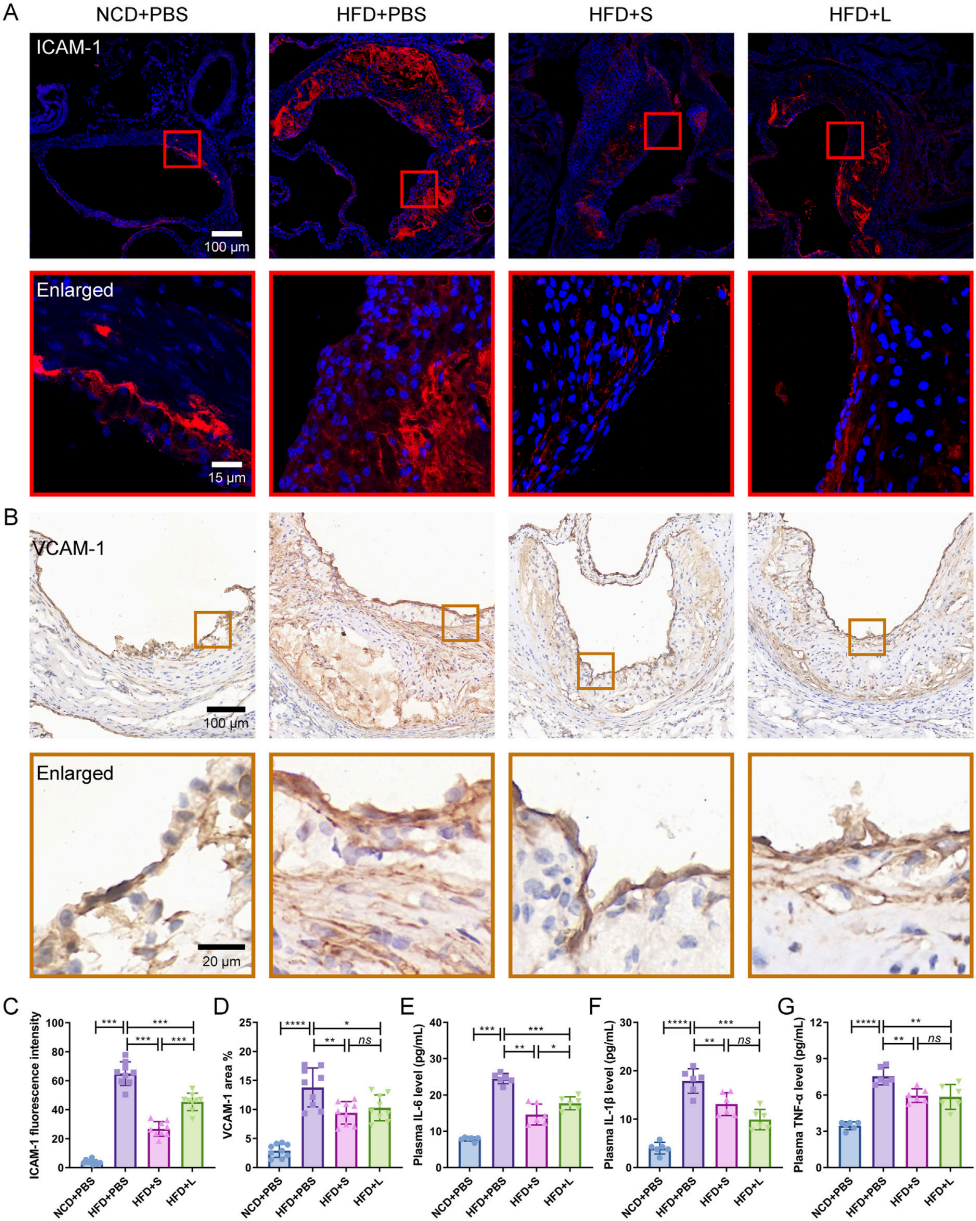
Inulin administration ameliorate localized and systemic inflammation in *ApoE* ^−/−^ mice. **(A)** Representative immunofluorescence staining for ICAM-1 (red) in atherosclerotic lesions. **(B)** Representative immunohistochemical staining for VCAM-1 (brown) in atherosclerotic lesions. **(C)** The fluorescence intensity of ICAM-1 was quantified by ImageJ software. **(D)** The positive area of VCAM-1 was quantified by ImageJ software and calculated as the percentage of total lesion area. **(E–G)** The circulating levels of interleukin-6 (IL-6), interleukin-1β (IL-1β) and tumor necrosis factor-α (TNF-α) were measured by ELISA. Data were shown as mean ± SD. *n* = 6 to 9 Significances were determined by one-way analysis of variance (ANOVA), followed by *post hoc* pairwise comparisons with the Tukey honest significant difference. *ns* (no significance), *P* < 0.05 (*), *P* < 0.01 (**), *P* < 0.001 (***).

Subsequently, the effects of inulin on localized and systemic inflammation were evaluated. ICAM-1 and VCAM-1 are two specific inflammation-related biomarker for AS, which indicated the localized inflammation. Immunofluorescence staining for ICAM-1 and immunohistochemical staining for VCAM-1 showed that HFD increased their expression level, while inulin decreased their contents in the atherosclerotic lesions area (Figures 3A–D). The effect of short-chain inulin in decrease the VCAM-1 content is more effective than the long-chain inulin (Figures 3A–D). To evaluation the systemic inflammation, the pivotal proinflammatory cytokines IL-6, IL-1β and TNF-αconcentrations in plasma were analyzed. Unsurprising, HFD treatment increased these cytokines, while inulin reversely decreased all these cytokines contents (Figures 3E–G), the short-chain inulin is more pronounced.

### 3.4 Inulin modulate intestinal barrier integrity

**FIGURE 4:**
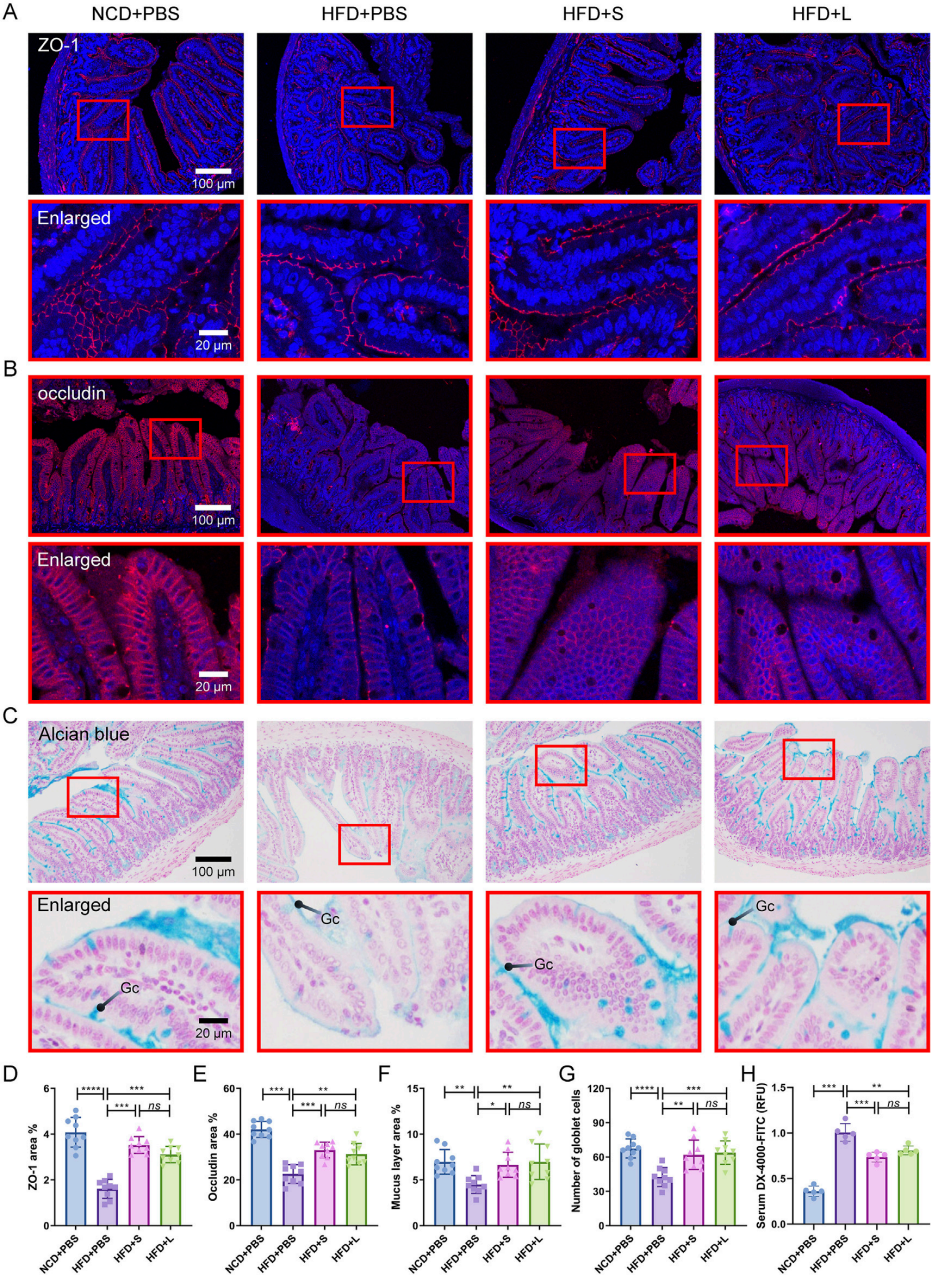
Inulin modulate intestinal barrier integrity in *ApoE* ^−/−^ mice. **(A)** Representative immunohistochemical staining for ZO-1 (red) in the colon. **(B)** Representative immunohistochemical staining for occludin (red) in the colon. **(C)** Representative Alcian blue staining of colon sections. The mucin layer and the goblet cells (Gc) were visualized under the microscope. **(D, E)** Quantitative analysis of images from **(A)** and **(B)**. **(F, G)** Quantitative analysis of the images from **(C)**. **(H)** Intestinal permeability was determined by measuring the 4,000 Da fluorescent dextran-FITC (DX-4000-FITC) level in serum.

Intestinal barrier integrity is critical for intestinal function, inflammatory and even AS. Thus, we first evaluated the intestinal epithelial barrier integrity by immunohistochemical staining for ZO-1 and Occludin, which are two biomarkers of intestinal epithelial barrier. The results showed that the HFD treatment decreased the expression of ZO-1 and Occludin, while inulin reinstated the expression profiles of ZO-1 and Occludin (Figures 4A, B, D, E). Furthermore, the spatial extent of the mucus sheath and the abundance of mucus-secreting goblet cells were ascertained through Alcian blue staining. Similarly, HFD treatment decreased the mucus overlay and goblet cell count, which was recovered by inulin treatment (Figures 4C, F, G). Lastly, an *in vivo* assessment of gut barrier integrity was conducted by analysis the translocation of FITC-dextran from the colonic milieu to the circulatory system. The data indicated that HFD treatment significantly increased FITC-dextran contents in the serum, which was pronounced reduction after the inulin treatment (Figure 4H). For these indexes of intestinal barrier integrity, the short-chain and long-chain inulin has no significant difference. Together, inulin protected the intestinal barrier integrity.

### 3.5 Inulin modify gut microbial diversity and functionality

**FIGURE 5:**
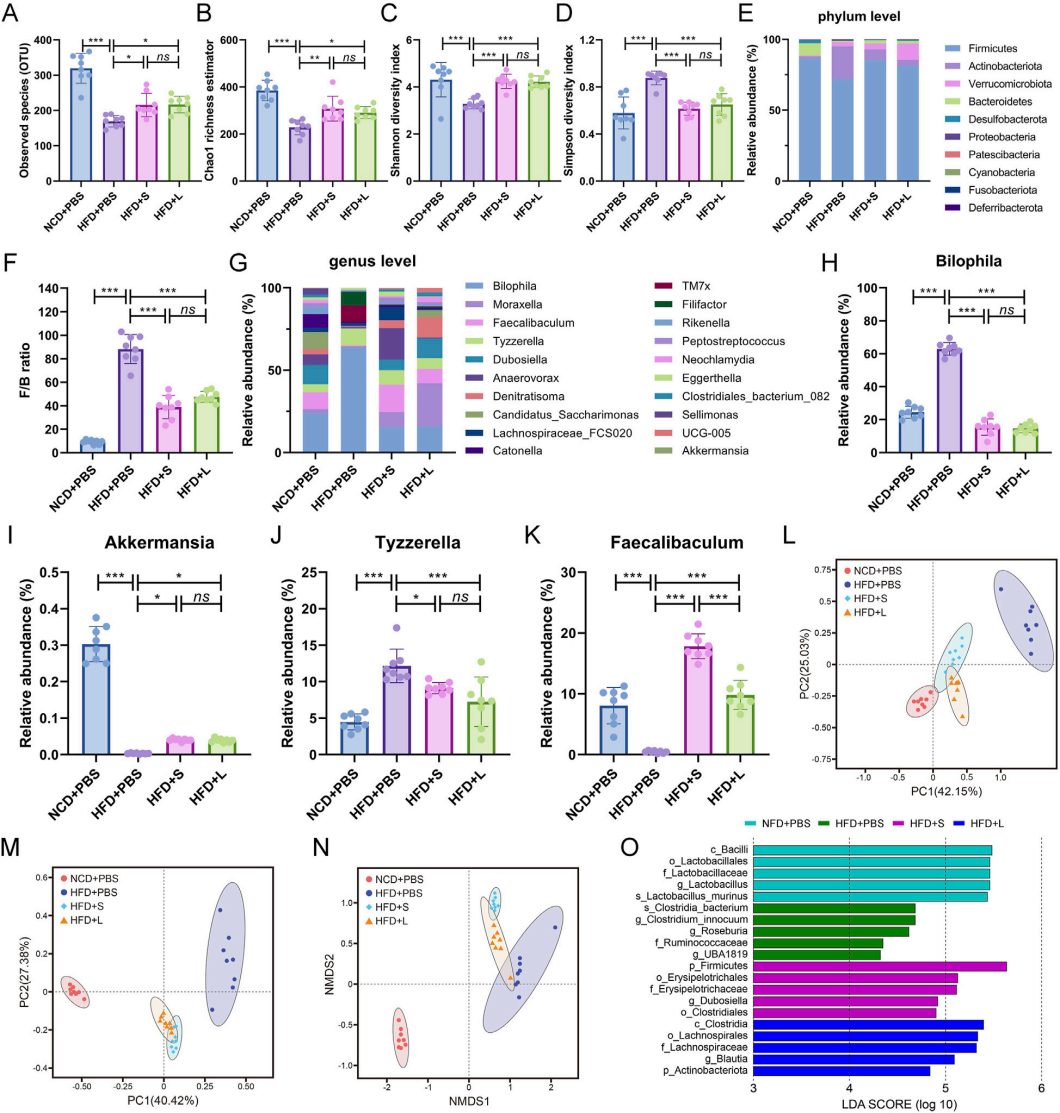
Inulin regulate gut microbial community in *ApoE* ^−/−^ mice. **(A)** Observed species. **(B)** Chao1 richness estimator. **(C)** Shannon diversity index. **(D)** Simpson diversity index. **(E)** Mean relative abundance of top 10 phyla. **(F)** Firmicutes/Bacteroidetes ratio. **(G)** Top 20 genera with mean relative abundance. The relative abundance of **(H)** Bilophila, **(I)** Akkermansia, **(J)** Tyzzerella, **(K)** Faecalibaculum. **(L)** The principal components analysis (PCA). **(M)** The principal coordinate analysis (PCoA). **(N)** The nonmetric multidimensional scaling (NMDS) index. **(O)** LEfSe histograms. Bacterial taxa that met the criterion of an LDA score > 4 were considered biomarker taxa. Data were shown as mean ± SD. *n* = 8. Significances were determined by one-way analysis of variance (ANOVA), followed by *post hoc* pairwise comparisons with the Tukey honest significant difference. *ns* (no significance), *P* < 0.05 (*), *P* < 0.01 (**), *P* < 0.001 (***).

To analysis the relationship between gut microbiota and atherosclerosis, the effect of inulin on gut microbial architecture is further investigated. The 16S rRNA high-throughput sequencing results showed that the HFD treatment significantly decreased the observed species (OUT) while the inulin administration rescued the observed species (Figure 5A). The community species richness (Chao 1 index) and community diversity (Shannon and Simpson indexes) represented the alpha diversity. The results showed that HFD treatment resulted the decline of Chao1 index (Figure 5B) and Shannon index (Figure 5C) but increased Simpson index (Figure 5D), while inulin reversed these indexes. To further analyze the structure and composition of gut microbiota, beta diversity based on the principal components analysis (PCA), principal coordinate analysis (PCoA), and nonmetric multidimensional scaling (NMDS) were performed. The PCA (Figure 5L) and PCoA (Figure 5M) analysis showed a relatively separate clustering between the HFD + PBS and other groups. Intriguingly, the NMDS analysis proffered overlapping realms between HFD + L and HFD + PBS (Figure 5N), suggesting microbial structural homogeneity between the two groups. These results indicated that HFD diet decreased the richness and diversity of bacterial communities while inulin treatment could keep the bacterial community’s homeostasis.

The abundance of bacteria also changed at the phylum and genus levels among different groups. The relative proportions of the top 10 gut microbiota at the phylum level were analyzed (Figure 5E), and the *Firmicutes*/*Bacteroidetes* (F/B) ratio was particular increased by HFD while decreased by inulin treatment (Figure 5F). For the deeper genus level analysis (Figure 5G), inulin administration declined the relative abundance of pathogen *Bilophila* (Figure 5H) and *Tyzzerella* (Figure 5J), which increased by HFD treatment. Conversely, the HFD treatment decreased the abundance of probiotic *Akkermansia* (Figure 5I) and *Faecalibaculum* (Figure 5K) while rescued by the inulin administration. Lastly, the linear discriminant analysis effect size (LEfSe) was performed on the different microbial features to identify the predominant bacteria in different groups. For example, at the family level, LEfSe analysis indicated that *Ruminococcaceae* had higher abundance in HFD group mice, the short-chain inulin group was characterized by a higher abundance of *Erysipelotrichaceae*, but the long-chain inulin group had predominant *Lachnospiraceae* (Figure 5O). Thus, inulin modified the gut microbial diversity and functionality.

## 4 Discussion

The present study systematically investigated the effect of different polymerization degrees of inulin on atherosclerosis and its related mechanisms. Our results indicated that inulin supplementation, especially the short-chain inulin, alleviated the progression of atherosclerotic plaques in HDF-fed *ApoE* ^−/−^ mice. This benefits of inulin in managing atherosclerosis are associated with its effects on regulating lipid metabolism, reducing inflammatory responses, enhancing intestinal barrier structure and function, and optimizing gut microbiota composition.

The effect of inulin on atherosclerosis is controversial. In this study, we demonstrated that inulin supplementation alleviated the progression of atherosclerotic plaques, which is consistent with some previous studies (Rault-Nania et al., 2006; Amato et al., 2017) but contradicts other reports (Hoving et al., 2018a; Hoving et al., 2018b). The different effects of inulin on atherosclerosis may be attributed to the atherosclerosis model used. Our study employed *ApoE* ^−/−^ mice fed a HFD, while other studies used different models, such as *ApoE* ^−/−^ mice fed sucrose-based diet (Rault-Nania et al., 2006), APOE*3-Leiden (E3L) mice fed a high cholesterol diet (Hoving et al., 2018a; Hoving et al., 2018b). *ApoE* ^−/−^ mice completely lack the APOE gene, whereas E3L mice express a variant of APOE; these differences result in distinct lipid metabolism and inflammatory responses, which are critical factors in atherosclerosis (Hoving et al., 2018b; Grainger et al., 2004). Clearly, inulin showed beneficial effects in alleviating atherosclerosis in the *ApoE* ^−/−^ mice fed a HFD model rather than in the E3L mice. Thus, it’s reasonable to propose that the varying effects of inulin on atherosclerosis are related to differences in lipid metabolism and inflammation responses in different mouse models.

The lipid metabolism disorder is a primary factor in the progression of atherosclerosis (Duan et al., 2023). In the present study, inulin (especially the short-chain inulin) treatment decreased plasma total triglyceride and low-density lipoprotein (LDL) levels, partly consistent with previous results in both mice (Rault-Nania et al., 2006) and humans (Russo et al., 2008; Lin et al., 2014). Inulin also alleviated the lipid deposit in liver adipocytes, similar to findings in other mouse study (Chen et al., 2023). The effect of inulin in improving lipid metabolism is agreed with most previous studies. However, the molecular mechanism is seldom explored. It’s known that sterol regulatory element binding protein 1 (SREBP1) involved in the *de novo* lipogenesis in liver (Dong, 2023). In this study, inulin (especially the short chain inulin) decreased HFD-induced high plasma total triglyceride levels and significantly downregulated SREBP1 mRNA expression liver tissues, suggesting that inulin may through inhibit lipogenesis to decrease total triglyceride by reducing SREBP1 expression. Additionally, the proprotein convertase subtilisin/kexin type 9 (PCSK9) plays a crucial effect in LDL metabolism by interacting with hepatic LDL receptors (LDLR) (Wu et al., 2012). In our study, the short-chain inulin (rather than long-chain inulin) decreased HFD-induced high LDL levels and reduced PCSK9 mRNA expression, suggesting that inulin might reduce plasma LDL content by downregulating liver PCSK9 expression. For cholesterol metabolism, cytochrome P450 family 7 subfamily A member 1 (CYP7A1), and ATP-binding cassette (ABC) transporter family members (ABCA1, ABCG1 and ABCG5) are key factors. Previous studies have shown that CYP7A1 restores cholesterol homeostasis(Yang T. et al., 2023); ABCA1 and ABCG1 reduce macrophage cholesterol accumulation, and ABCG5 promotes cholesterol elimination from the body via hepatobiliary secretion (Oram and Vaughan, 2006). In this study, inulin (especially the short chain form) tended to decrease HFD-induced high plasma cholesterol content while upregulate mRNA expression of CYP7A1, ABCA1 and ABCG1. All these information suggest that inulin decrease cholesterol content may through activating CYP7A1 and ABC family members (ABCA1, ABCG1). Together, inulin may through regulating SREBP1, PCSK9, CYP7A1, ABCA1 and ABCG1 to maintain lipid metabolism homeostasis.

Atherosclerosis is now understood as a long-term immune-mediated inflammatory condition (Meng et al., 2023). In this study, inulin, particularly short-chain inulin, decreased HFD-induced high levels of proinflammatory cytokines such as IL-6, IL-1β, and TNF-α in plasma, indicating that inulin administration could ameliorate systemic inflammation, consistent with previous report (Wan et al., 2020). Inulin, as a non-digestible carbohydrate, primarily exerts immunomodulatory effects within the intestine (Akram et al., 2019). However, it is unclear whether inulin could regulate inflammation at atherosclerotic lesions. Intercellular adhesion molecule-1 (ICAM-1) is a member of the immunoglobulin (Ig) superfamily and is constitutively present on endothelial cells, which is increased in the atherosclerotic tissue (Lawson and Wolf, 2009). Similarly, the vascular cell adhesion molecule-1 (VCAM-1) is also critical for the atherosclerotic processes, especially in foam cell formation (Malekmohammad et al., 2019). In our study, ICAM-1 and VCAM-1 levels increased in the atherosclerotic lesions after HFD-fed *ApoE* ^−/−^ mice, while inulin (especially short-chain inulin) decreased these levels in atherosclerotic lesions. This indicates that inulin also ameliorates localized inflammation at the atherosclerotic lesions area.

Inulin-type fructans are widely regarded as prebiotics for their ability to be selectively utilized by gut microbiota to confer health benefits (Hughes et al., 2022). The present study’s sequencing results showed that inulin alleviated HFD-induced low microbiome diversity, as indicated by the observed species, Chao1 index, and Shannon index, suggesting that inulin administration improved the richness and diversity of bacterial communities. At the phylum level, the most various gut microbiota were *Firmicutes* and *Bacteroidetes*, the high *Firmicutes*/*Bacteroidetes* (F/B) ratio could be used a biomarker of gut dysbiosis in obese patients (Magne et al., 2020). In this study, the ratio of F/B was elevated significantly by the HFD diet, which decreased by the inulin supplement. Furthermore, at the genus level, *Akkermansia* and *Faecalibaculum* has been reported to be beneficial in improving host metabolic disorders and inflammation (Luo et al., 2022; Wang et al., 2020). It’ reported that HFD diet could enrich *Bilophila* and *Tyzzerella*, which are associated with inflammation and lipid metabolism (Zhao et al., 2022). In our present study, inulin supplement rescued HFD-lowed amount of *Akkermansia* and *Faecalibaculum*, while inulin decreased HFD-induced high counts of *Bilophila* and *Tyzzerella*. Interestingly, there were no difference between the short and long chain inulin among the microbiota diversity or at the phylum level, while short-chain inulin especially increased the probiotics *Faecalibaculum* much more than the long-chain form. Thus, the above information suggested that inulin administration improved the gut microbiota, especially the probiotics. However, the relationship among inulin, gut microbiota and atherosclerosis is not clear. Recent report indicated that reduction in atherosclerotic plaque was associated with increases in bacterial diversity, reduction in the F/B ratio, and upregulation of *Akkermansia* (Centner et al., 2023). *Akkermansia* is one of the most widely researched probiotics, has been reported to be of great value in improving host metabolic disorders, inflammation and immune responses (Luo et al., 2022). Moreover, study in ApoE^−/−^ mice found that replenishment with *Akkermansia* reversed Western diet-induced exacerbation of atherosclerotic lesion formation by preventing metabolic endotoxemia-induced inflammation and restoration of the gut barrier (Li et al., 2016). Thus, it’s reasonable to speculated that diet inulin reduced HFD-induced atherosclerosis may be partly attributed to enhanced gut microflora diversity and probiotics abundance, which could improve metabolism, immune and gut barrier.

Besides gut microflora, the intestinal barrier is also important for health and atherosclerosis (Lewis and Taylor, 2020). The intestinal barrier is composed of many subunits, including the intestinal mucosa and the epithelium barrier (Lewis and Taylor, 2020). Previous studies demonstrated that inulin supplementation restored the intestinal epithelium barrier by up-regulating expressions of tight junction proteins (Yang Z. et al., 2023; Beisner et al., 2021), which is consistent with our present study. However, the effect of inulin on intestinal mucosa is largely unclear. Intestinal mucosa act as a barrier between luminal contents and underlying tissue, of which the goblet cells secreted mucins play critical effect (Lewis and Taylor, 2020). Our present study indicated that inulin improved the intestinal mucosa function as shown by the lowed intestinal permeability, increased mucin layer area and the goblet cells number. Additionally, the effect of short-chain and long-chain inulin on the intestinal barrier have no significant different. Thus, the inulin administration protects the intestinal barrier by improving both intestinal mucosa and epithelium tight junction.

## 5 Conclusion

This study systematically investigated the effects of two types of inulin on atherosclerosis and the potential mechanisms involving lipid metabolism, inflammation, gut microbiota, and the intestinal barrier. Our results demonstrate that inulin administration alleviated the progression of atherosclerosis. This is tightly associated with inulin’s beneficial efficacy in modulating lipid metabolism, attenuating inflammatory responses, and optimizing gut microbiota composition. Furthermore, short-chain inulin was found to be more effective than its long-chain form. Thus, the present study provides robust evidence for dietary inulin intervention’s potential in treating atherosclerosis and potentially other cardiovascular diseases.

## Data Availability

The data presented in the study are deposited in the NCBI repository, accession number PRJNA1166712. https://www.ncbi.nlm.nih.gov/bioproject/PRJNA1166712.

## Abbreviations

AS, Atherosclerosis; ELISA, enzyme-linked immunosorbent assay; HFD, high fat diet; HDL, high-density lipoprotein; ICAM-1, intercellular adhesion molecule-1; IL-1β, interleukin 1β; LDL, low-density lipoprotein; NCD, normal chow diet; ORO, Oil Red O staining; TC, total cholesterol; TG, total triglyceride; TNF-α, tumor necrosis factor-α; VCAM-1, vascular cell adhesion protein 1; ZO-1, zonula occludens-1.
